# Methodological Approaches for Ultrasound Assessment of Peripheral and Abdominal Muscle Thickness in Intensive Care: A Scoping Review and Proposed Reporting Framework

**DOI:** 10.1002/pri.70303

**Published:** 2026-08-08

**Authors:** Pedro Vinicius Porfirio, Wagner Souza Leite, Layane Santana Pereira Costa, Pedro Henrique de Moura, Ana Célia Oliveira dos Santos, Emanuel Fernandes Ferreira da Silva Junior, Elaine Araújo de Souza, Marismar Fernandes do Nascimento, Helga Cecilia Muniz de Souza, Shirley Lima Campos

**Affiliations:** ^1^ Department of Physiotherapy Federal University of Pernambuco (UFPE) Recife Pernambuco Brazil; ^2^ Department of Physical Therapy Graduate Program in Rehabilitation Science University of Florida Gainesville Florida USA; ^3^ University of Pernambuco (UPE) Recife Pernambuco Brazil

**Keywords:** critical illness, intensive care units, muscle thickness, ultrasonography

## Abstract

**Objective:**

To map and synthesize methodological approaches used for ultrasound (US) assessment of peripheral (upper‐ and lower‐limb) and abdominal muscle thickness in critically ill patients, and to propose a preliminary Standard Operating Procedure (SOP) as a structured reporting framework.

**Methods:**

A review was conducted following the Preferred Reporting Items for Systematic Reviews and Meta‐Analyses Extension for Scoping Reviews checklist, using systematic searches on PubMed, BIREME (including MEDLINE, LILACS and IBECS) and Embase. Two reviewers independently screened, selected, and extracted data from studies published between 2015 and 2025. Extracted data included muscle groups assessed, thickness measurement criteria, patient and probe positioning, operational US parameters, and reliability information. The proposed SOP was developed based on recurring methodological patterns identified in the literature and subsequently refined through external expert review and pilot feasibility testing in ICU settings.

**Results:**

Of 2.293 identified records, 29 studies (1.736 patients) were included. B‐mode imaging and linear transducers were the most frequently reported US parameters, and the supine position with the head of the bed elevated to 30° was the predominant patient position. Although 79.3% of studies described muscle thickness measurement criteria, important methodological details such as depth, gain, and anatomical landmarks were inconsistently reported. Lower‐limb muscles were the most frequently assessed, whereas upper‐limb and abdominal muscles remained comparatively underrepresented. Considerable variability was identified across acquisition procedures, anatomical landmarks, measurement criteria, and reporting practices.

**Conclusions:**

Ultrasound assessment of muscle thickness in critically ill patients shows substantial methodological and reporting variability. A preliminary SOP was developed and pilot‐tested to improve consistency and comparability. Standardization may support bedside monitoring and inform physiotherapy assessment and rehabilitation decisions, although multicenter validation is required.

**Trial Registration:**

Open Science Framework: https://doi.org/10.17605/OSF.IO/S95MJ

## 1 Introduction

Bedside ultrasound (US) has become an essential point‐of‐care imaging modality in intensive care units (ICUs), offering a radiation‐free and non‐invasive approach for detecting morphological and functional changes across different organs and systems (Bald et al. 2026; Gu et al. 2025). Among the multiple US applications, muscle ultrasonography has gained prominence in the literature for monitoring muscle‐wasting trajectories, guiding therapeutic interventions, and serving as a predictor of clinical outcomes (Bald et al. 2026; Lau and See 2022). Muscle US assessment provides clinically relevant information to support personalized rehabilitation planning, monitor muscle loss and functional decline throughout the ICU stay, and help identify ICU‐acquired weakness—a condition associated with prolonged mechanical ventilation, long‐term physical disability, and increased morbi‐mortality (De Rosa et al. 2023; Wang et al. 2020). These findings may guide mobilization strategies to either mitigate or prevent those adverse outcomes (Lima et al. 2024; Santos de Arruda et al. 2025; Saraiva et al. 2025).

While previous reviews have explored ultrasound‐based muscle assessment in critically ill patients, they have predominantly focused on specific muscle groups (Formenti et al. 2019; Lau and See 2022; Nascimento et al. 2021; Venco et al. 2024), with comparatively less attention given to abdominal (Formenti et al. 2019; Nascimento et al. 2021) and upper‐limb muscles (Venco et al. 2024), as well as to methodological consistency across anatomical sites. As a result, substantial heterogeneity persists in US acquisition protocols, anatomical landmark definitions, patient positioning, and measurement techniques, limiting reproducibility and comparability across studies. No prior review has comprehensively synthesized methodological approaches across both peripheral (upper‐ and lower‐limb) and abdominal muscles while explicitly addressing reporting variability and its implications for standardization.

Although efforts to standardize muscle ultrasound assessment have been undertaken in other clinical settings, ICU‐specific methodological recommendations remain limited. The SARCUS working group published consensus recommendations (Perkisas et al. 2019, 2021) for standardized US assessments of limb muscles in individuals with sarcopenia. However, sarcopenic conditions encountered in the ICU frequently overlap with ICU‐acquired weakness and do not affect muscles uniformly, as abdominal, upper‐, and lower‐limb muscles present distinct biomechanical functions, patterns of activation, susceptibility to disuse, and potential trajectories of atrophy and recovery (Le Stang et al. 2024; Ozdemir et al. 2022). Abdominal muscles are essential for respiratory mechanics, airway defense, and core stability, and their deterioration can prolong ventilator dependence and compromise functional recovery (Amara et al. 2022; Kawahara et al. 2020; Yang et al. 2021). Upper limb muscles contribute to transfers, bed mobility, task performance, and patient interaction while hospitalized, and their atrophy has been associated with in‐hospital mortality and impaired physical function in critically ill adults (Nakanishi, Oto, et al. 2020; Nakanishi, Takashima, et al. 2020). Consequently, assessment focused on isolated muscle groups may fail to fully characterize muscle impairment and functional decline, potentially limiting rehabilitation‐oriented decision‐making and individualized exercise prescription.

Therefore, this scoping review aimed to provide a comprehensive synthesis of US‐based methods for assessing muscle thickness across upper‐limb, lower‐limb, and abdominal muscle groups in critically ill patients. Specifically, this review identified the most frequently reported assessment protocols, compared methodological variations and technical considerations across muscle groups, and proposed a structured preliminary Standard Operating Procedure (SOP) for standardized reporting of US acquisition and assessment procedures. Rather than prescribing a single acquisition technique or muscle‐specific assessment protocol, the proposed SOP was designed as a reporting framework intended to promote transparent documentation of methodological decisions, settings, and measurement procedures across different assessment approaches. By addressing current reporting inconsistencies, this review may support improved reproducibility, comparability, and interpretation of muscle US findings in critical care practice and research. To our knowledge, this is the first review to integrate methodological evidence across multiple muscle groups and translate it into a structured reporting framework for muscle ultrasound assessment in critically ill patients.

## 2 Methods

This scoping review is registered with the Open Science Framework (https://doi.org/10.17605/OSF.IO/S95MJ) and follows the recommendations of the Joanna Briggs Institute (JBI), according to the JBI Reviewers Manual—Methodology for Scoping Reviews and the most recent guidance (Peters et al. 2020). The scoping review framework included five stages: (1) identifying the research questions; (2) identifying relevant studies; (3) study selection; (4) charting the data; and (5) collating, summarizing and reporting the results (Pollock et al. 2023). The results were based on the Preferred Reporting Items for Systematic Reviews and Meta‐Analysis Extension for Scoping Reviews checklist (Supporting Information S1: Table S1) (Tricco et al. 2018).

### 2.1 Search Strategy

A systematic literature search was conducted from July 1, 2025 to October 30, 2025, using the following databases: PubMed, BIREME (including MEDLINE, LILACS and IBECS) and Embase, with the complete database‐specific search strings provided in Supporting Information S1. PubMed and Embase were prioritized for their broad coverage of medical and intensive care research, while BIREME was included to improve the retrieval of studies indexed in MEDLINE, LILACS, and IBECS. Search terms were developed using Health Sciences Descriptors (DeCS) and Medical Subject Headings (MeSH) descriptors, combined with free‐text keywords related to critical illness, intensive care, ultrasonography, skeletal muscle, muscle thickness, and peripheral musculature, and adapted to the indexing structure and syntax of each database. References of the included studies were manually screened to identify additional eligible publications. Gray literature and preprint servers were excluded due to limited accessibility, inconsistent reporting standards, and the absence of formal peer review. Because the primary aim of this scoping review was to synthesize ultrasound assessment protocols and inform a preliminary SOP for standardized reporting, we selected databases known to index methodological and clinical studies relevant to ICU muscle ultrasound.

### 2.2 Eligibility Criteria

Inclusion criteria were as follows: (1) publications with critically ill adult patients (age ≥ 18 years) of either sex, regardless of pathology or characteristics; (2) included techniques and protocols for using US to measure abdominal, upper, and/or lower limb muscle thickness in the intensive care unit; and (3) published after January 1, 2015.

The review specifically focused on adult ICU populations because critically ill patients present unique physiological and clinical characteristics, including fluid shifts, mechanical ventilation, sedation, immobility, and rapid muscle wasting that affect US acquisition, image interpretation, and the feasibility of standardized assessment protocols. These ICU‐specific factors are not present in outpatient, rehabilitation, athletic, or general hospitalized populations, making their ultrasound methodologies insufficiently comparable for developing ICU‐focused procedural guidance. Therefore, studies conducted exclusively in non‐ICU or mixed non‐critically ill cohorts were excluded to ensure that the synthesized protocols and the proposed preliminary SOP were directly applicable to the ICU setting.

Exclusion criteria were as follows: (1) literature types including reviews (e.g., scoping, systematic, or narrative reviews), research protocols, conference abstracts, case reports, book reviews, letters to the editor, editorials, and studies published exclusively in preprint servers without peer review; and (2) studies that did not provide original methodological data for US assessment protocols, including duplicate publications, secondary analyses, or subsequent reports derived from cohorts and protocols already described in previously included studies.

### 2.3 Study Selection and Data Extraction

Title/abstract and full‐text screening, study selection, and data extraction were conducted independently by two reviewers (P.V.P., W.S.L.) using predefined eligibility criteria. Any disagreements at either the screening or data‐extraction stages were resolved through discussion, and when consensus could not be reached, a third reviewer (S.L.C.) adjudicated the final decision. Prior to formal screening, a calibration exercise using a random sample of 10 studies was conducted to ensure consistent interpretation of eligibility criteria. Any disagreements during screening or data extraction were resolved through discussion, with a third reviewer adjudicating unresolved cases. Data extraction was carried out independently using a standardized, piloted extraction form, followed by cross‐checking to ensure accuracy and completeness.

The extracted variables were defined through consensus among the research team and aligned with the terminology used across the included studies. Extracted data included authors, year of publication, country of origin, study objectives, study design, participant characteristics, muscle groups or anatomical regions assessed, reliability data, and methodological US parameters, including imaging mode, transducer type, depth, frequency, thickness criteria, body positioning, and probe positioning. The results summary is presented in both the main manuscript and Supporting Information S1.

### 2.4 Development of the Standard Operating Procedure

**FIGURE 1:**
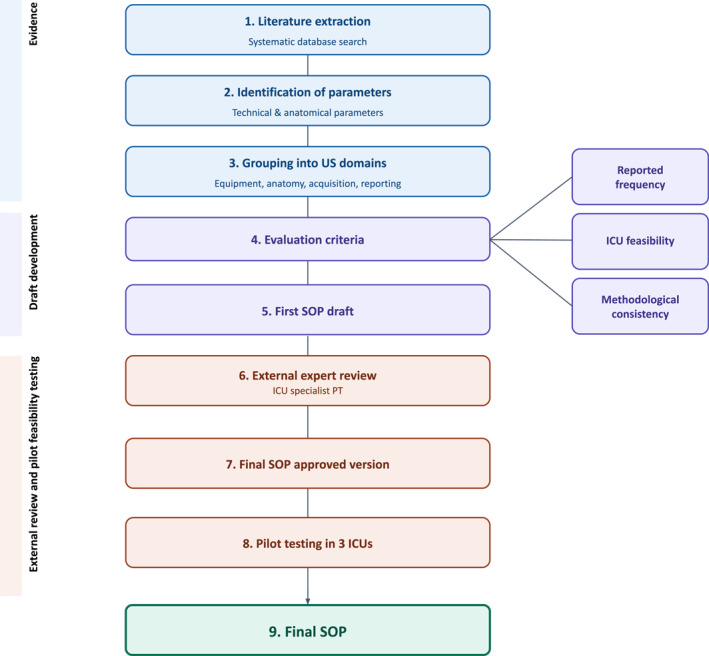
Workflow for the development of the standard operating procedure for standardized reporting of ultrasound acquisition and assessment procedures. ICU, intensive care unit; PT, physiotherapist; SOP, standard operating procedure; US, ultrasound.

The SOP was developed through a structured multistep process based on the methodological heterogeneity identified during data extraction (Figure 1). Initially, all US assessment parameters reported in the included studies were cataloged and grouped into the following methodological domains: muscle selection, patient positioning, probe positioning, transducer characteristics, image acquisition parameters, and thickness measurement criteria. Subsequently, each parameter was evaluated according to three predefined criteria: (1) frequency of reporting across studies, (2) methodological consistency between studies, and (3) feasibility and applicability in critically ill patients and ICU environments. Importantly, frequently reported parameters were not automatically considered best practices. Instead, parameter selection was informed by reporting frequency together with alignment with recommendations and technical considerations described in the musculoskeletal and critical care US literature.

Three independent external experts (P.H.d.M., H.C.M.d.S., E.A.d.S.) with experience in musculoskeletal US and critical care independently reviewed the preliminary SOP and provided feedback regarding methodological consistency, clinical relevance, clarity of operational definitions, and feasibility across different ICU settings. Experts were selected according to predefined criteria, including at least 5 years of clinical experience with US assessment in ICU, recognized specialist certification granted by the Brazilian Federal Council of Physical Therapy and Occupational Therapy, and professional affiliation with different hospital institutions to ensure diversity of clinical perspectives. Suggested modifications were discussed in virtual meetings and considered for incorporation into the final draft, and the final version was approved by all reviewers.

The final SOP underwent pilot feasibility testing in three general ICU to assess its real‐world applicability as a reporting framework. The pilot evaluation focused on usability, clarity of procedural instructions, completeness of documentation, and feasibility of implementation during routine ICU workflows. As the SOP was designed as a reporting framework rather than a measurement protocol, the pilot testing did not aim to generate outcome data, but rather to evaluate feasibility and usability in routine clinical practice.

## 3 Results

**FIGURE 2:**
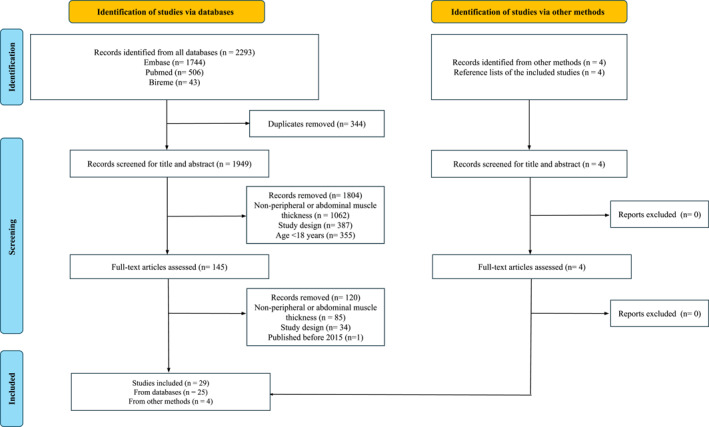
PRISMA 2020 flow diagram study selection process, including searches of databases, registers, and other sources.

After screening and consensus among examiners, 29 studies were included to describe US methodologies for assessing thickness in the upper‐limb (Supporting Information S1: Table S2), lower‐limb (Supporting Information S1: Table S3) and abdominal (Supporting Information S1: Table S4) muscle groups (Figure 2). These studies cumulatively involved 1.736 adult participants and they addressed several objectives, including evaluating therapeutic effects, monitoring muscle wasting during ICU stay, evaluating diagnostic accuracy, analyzing intra‐ and/or inter‐examiner reliability or examining associations with clinical outcomes, such as in‐hospital mortality, prediction of weaning success, physical function, frailty or nutritional risk (Supporting Information S1: Table S5).

**FIGURE 3:**
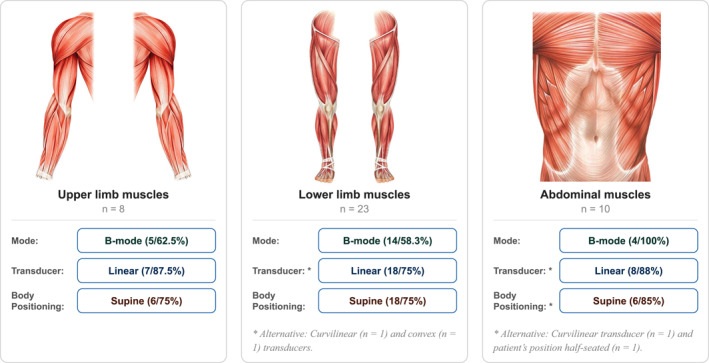
Illustrated muscle evaluation methodology comparison (*n*/%—relative frequency of studies that described technical standards for ultrasound assessment). Illustration adapted from sciencephotogallery.com. B‐mode, brightness mode.

Some methodological patterns and gaps have emerged across the included studies. Lower‐limb muscles were assessed most frequently (*n* = 23/29), followed by abdominal muscles (*n* = 10/29) and upper limbs (*n* = 8/29), which are clinically important for functional recovery, but underrepresented in the literature. Across muscle groups, B‐mode imaging and linear transducers were the most commonly reported US parameters, and patient positioning favored the supine position with the head of the bed elevated to approximately 30° (Figure 3). Upper‐limb and abdominal muscle assessments demonstrated greater methodological consistency in mode and transducer selection, whereas lower‐limb protocols showed greater heterogeneity.

Frequency ranges varied between 4 and 13 MHz for upper limb, 4–13 MHz for lower limbs and 4–12 MHz for abdominal muscles, which may reflect the depth of muscle groups of interest in each body segment (Barbosa et al. 2023; Chapple et al. 2022; Dall’ Acqua et al. 2017; Dams et al. 2024; de Moura et al. 2023; Fritsch et al. 2024; Haaksma et al. 2022; Hadda et al. 2018; Lambell et al. 2021; Li et al. 2020; Mayer et al. 2020; Palakshappa et al. 2018; Rajagopal et al. 2023; Sarwal et al. 2015; Silva‐Gutiérrez et al. 2023; Sundarsingh et al. 2025; Toledo et al. 2021; Vishwas et al. 2023; Witteveen et al. 2017; Yang et al. 2021; Zhang et al. 2021). Despite the frequent use of linear probes, inconsistent reporting of transducer frequency, acquisition depth, and image optimization parameters may substantially affect the reproducibility of these techniques for accurately visualizing the deeper muscle layers, particularly in abdominal assessments and in patients with edema or obesity. Only six studies reported all assessed methodological parameters (Table 1) (Barbosa et al. 2023; Dall’ Acqua et al. 2017; de Moura et al. 2023; Hadda et al. 2018; Lambell et al. 2021; Toledo et al. 2021).

**TABLE 1:** Ultrasonography‐assessed muscle thickness reported parameters and patient‐related features.

Author (year)	Mode	Transducer	Depth	Frequency	Body positioning	Probe positioning	Criteria for thickness	Total (yes, *n* = 7)
Santos de Arruda et al. (2025)^U|L^	N	Y	N	N	Y	Y	N	3
Sundarsingh et al. (2025)^U|L^	N	Y	N	Y	Y	Y	Y	5
Dams et al. (2024)^L^	N	Y	Y	Y	Y	Y	N	5
Fritsch et al. (2024)^L^	Y	Y	N	Y	N	Y	Y	5
Gürsoy et al. (2023)^L|A^	N	Y	N	N	Y	Y	Y	4
Nguyen et al. (2023)^L|A^	N	Y	N	N	Y	Y	N	3
Rajagopal et al. (2023)^L^	Y	Y	N	Y	Y	Y	Y	6
Barbosa et al. (2023)^U|L^	Y	Y	Y	Y	Y	Y	Y	7
de Moura et al. (2023)^U|L|A^	Y	Y	Y	Y	Y	Y	Y	7
Silva‐Gutiérrez et al. (2023)^L^	N	Y	N	Y	Y	Y	Y	5
Vishwas et al. (2023)^A^	N	Y	N	N	N	Y	N	2
Amara et al. (2022)^A^	N	Y	N	N	N	Y	N	2
Haaksma et al. (2022)^A^	N	Y	Y	Y	Y	Y	Y	6
Chapple et al. (2022)^U|L^	Y	N	Y	Y	Y	Y	N	5
Andrade‐Junior et al. (2021)^L^	Y	N	N	N	N	Y	Y	3
Lambell et al. (2021)^U|L|A^	Y	Y	Y	Y	Y	Y	Y	7
Toledo et al. (2021)^L^	Y	Y	Y	Y	Y	Y	Y	7
Yang et al. (2021)^A^	Y	Y	N	Y	Y	Y	Y	6
Zhang et al. (2021)^L^	N	Y	N	Y	Y	Y	N	4
Li et al. (2020)^L^	N	Y	N	Y	Y	Y	Y	5
Mayer et al. (2020)^U|L^	Y	Y	Y	Y	N	Y	N	5
McCaughey et al. (2019)^A^	N	N	N	N	N	Y	N	1
Hadda et al. (2018)^U|L^	Y	Y	Y	Y	Y	Y	Y	7
Palakshappa et al. (2018)^L^	N	Y	N	Y	N	Y	Y	4
Dall’ Acqua et al. (2017)^A^	Y	Y	Y	Y	Y	Y	Y	7
Witteveen et al. (2017)^U|L^	N	Y	Y	Y	N	Y	Y	5
Paris et al. (2017)^L^	Y	Y	N	N	Y	Y	Y	5
Parry et al. (2015)^L^	N	Y	N	Y	Y	Y	N	4
Sarwal et al. (2015)^L^	N	Y	Y	Y	Y	Y	Y	6
Total (yes, *n* = 29)	13	26	12	21	21	29	19	—

Abbreviations: ^A^, studies assessing abdominal muscle thickness; ^L^, studies assessing lower‐limb muscle thickness; N, no reported; ^U^, studies assessing upper‐limb muscle thickness; Y, yes reported.

The probe‐skin angle was predominantly described as perpendicular to the skin surface (Chapple et al. 2022; Dall’ Acqua et al. 2017; Dams et al. 2024; Fritsch et al. 2024; Haaksma et al. 2022; Hadda et al. 2018; Lambell et al. 2021; Paris et al. 2017; Rajagopal et al. 2023; Sarwal et al. 2015; Sarwal et al. 2015; Sundarsingh et al. 2025; Toledo et al. 2021; Yang et al. 2021), transverse (short‐axis) scans (Barbosa et al. 2023; Gürsoy et al. 2023), or using non‐standard terms, such as “horizontal” (Amara et al. 2022; Vishwas et al. 2023) or “vertical” (Li et al. 2020) to describe probe orientation. Inconsistent terminology for probe orientation and acquisition planes may contribute to ambiguity in protocol replication and in the interpretation of muscle thickness measurements across studies.

Only seven studies explicitly specified acquiring images in the longitudinal (long‐axis) plane (Barbosa et al. 2023; Fritsch et al. 2024; Mayer et al. 2020; Palakshappa et al. 2018; Rajagopal et al. 2023; Sarwal et al. 2015; Zhang et al. 2021). Depth of image acquisition were unspecified in five studies (Dams et al. 2024; Haaksma et al. 2022; Lambell et al. 2021; Toledo et al. 2021; Witteveen et al. 2017), while reported values ranged from 3.5 to 7.4 cm (Barbosa et al. 2023; Chapple et al. 2022; Dall’ Acqua et al. 2017; de Moura et al. 2023; Hadda et al. 2018; Mayer et al. 2020; Sarwal et al. 2015). Gain for adjusting image brightness was poorly reported (*n* = 4/29) (Barbosa et al. 2023; Chapple et al. 2022; de Moura et al. 2023; Haaksma et al. 2022). Incomplete reporting of depth and gain parameters may directly affect image quality, muscle border delineation, and measurement reliability.

Regarding muscle thickness measurement, most studies provided a methodological description (Supporting Information S1: Tables S2–S4) (Barbosa et al. 2023; Dall’ Acqua et al. 2017; de Andrade‐Junior et al. 2021; de Moura et al. 2023; Fritsch et al. 2024; Gürsoy et al. 2023; Haaksma et al. 2022; Hadda et al. 2018; Lambell et al. 2021; Li et al. 2020; McCaughey et al. 2019; Palakshappa et al. 2018; Paris et al. 2017; Parry et al. 2015; Rajagopal et al. 2023; Sarwal et al. 2015; Silva‐Gutiérrez et al. 2023; Sundarsingh et al. 2025; Toledo et al. 2021; Vishwas et al. 2023; Witteveen et al. 2017; Yang et al. 2021; Zhang et al. 2021). However, the level of detail provided for measurement procedures varied substantially, particularly regarding thickness measurement criteria.

Seventeen studies provided figures of the images seen in the US (Amara et al. 2022; Barbosa et al. 2023; Dams et al. 2024; de Andrade‐Junior et al. 2021; Haaksma et al. 2022; Li et al. 2020; Mayer et al. 2020; Nguyen et al. 2023; Palakshappa et al. 2018; Paris et al. 2017; Parry et al. 2015; Sarwal et al. 2015; Sundarsingh et al. 2025; Toledo et al. 2021; Vishwas et al. 2023; Yang et al. 2021; Zhang et al. 2021); of these, two also brought figures of the positioning to the transducer (Barbosa et al. 2023; Yang et al. 2021).

Patient positioning was predominantly supine (*n* = 25/29) (Barbosa et al. 2023; Chapple et al. 2022; Dall’ Acqua et al. 2017; Dams et al. 2024; de Moura et al. 2023; Gürsoy et al. 2023; Haaksma et al. 2022; Hadda et al. 2018; Lambell et al. 2021; Li et al. 2020; Mayer et al. 2020; Nguyen et al. 2023; Paris et al. 2017; Parry et al. 2015; Rajagopal et al. 2023; Santos de Arruda et al. 2025; Sarwal et al. 2015; Silva‐Gutiérrez et al. 2023; Sundarsingh et al. 2025; Toledo et al. 2021; Vishwas et al. 2023; Yang et al. 2021; Zhang et al. 2021).

Only a minority of studies performed bilateral (Amara et al. 2022; de Moura et al. 2023; Gürsoy et al. 2023; Nguyen et al. 2023; Palakshappa et al. 2018; Parry et al. 2015; Santos de Arruda et al. 2025; Toledo et al. 2021; Yang et al. 2021; Zhang et al. 2021) or multi‐segment (abdominal, upper‐, and lower‐limb muscles) assessments (de Moura et al. 2023; Lambell et al. 2021), limiting comprehensive evaluation of muscle groups. This predominance of single‐site assessments may fail to fully characterize regional muscle involvement in critically ill patients, particularly given the potential heterogeneity of muscle wasting across body segments.

Evaluator training and operator expertise were inconsistently reported across studies (*n* = 18/29) (Amara et al. 2022; Barbosa et al. 2023; Chapple et al. 2022; Dall’ Acqua et al. 2017; de Moura et al. 2023; Fritsch et al. 2024; Gürsoy et al. 2023; Haaksma et al. 2022; Hadda et al. 2018; Lambell et al. 2021; Li et al. 2020; Mayer et al. 2020; Palakshappa et al. 2018; Parry et al. 2015; Santos de Arruda et al. 2025; Sarwal et al. 2015; Silva‐Gutiérrez et al. 2023; Sundarsingh et al. 2025; Toledo et al. 2021; Witteveen et al. 2017; Zhang et al. 2021) and reliability analysis (*n* = 11/29) (Amara et al. 2022; Barbosa et al. 2023; Dall’ Acqua et al. 2017; Dams et al. 2024; de Moura et al. 2023; Haaksma et al. 2022; Lambell et al. 2021; Mayer et al. 2020; Paris et al. 2017; Sarwal et al. 2015; Zhang et al. 2021). Studies assessing lower‐limb muscles more frequently reported reliability testing and operator expertise (*n* = 8) than those assessing upper‐limb (*n* = 5) and abdominal (*n* = 3) muscles, suggesting greater methodological robustness (Supporting Information S1: Table S5). This observation may reflect the longer history of lower‐limb ultrasound assessment in critically ill patients, particularly involving the quadriceps, which has been the focus of earlier investigations and dedicated evidence syntheses (Venco et al. 2024). In addition, the more frequent reporting of evaluator expertise and reliability analyses may indicate a comparatively higher degree of methodological refinement and standardization in lower‐limb protocols than in upper‐limb and abdominal muscle assessments.

Among the included studies, pilot assessments were conducted only for sample size calculation (Dall’ Acqua et al. 2017; Toledo et al. 2021) or intra‐rater reliability (de Moura et al. 2023; Mayer et al. 2020). Overall, incomplete reporting of evaluator training, reliability metrics, and image acquisition parameters limited assessment of methodological rigor and may affect interpretation and reproducibility of US‐derived muscle thickness measurements.

These findings collectively demonstrate substantial variability in US practice across acquisition, measurement, and reporting, which challenges reproducibility and cross‐study interpretation. Methodological inconsistencies were particularly evident in upper‐limb and abdominal muscle assessments, whereas quadriceps‐focused protocols showed comparatively greater standardization and reporting consistency. These recurrent gaps in measurement techniques underscore the need for a standardized framework that improves methodological reporting and facilitates comparison across studies and muscle groups.

The developed SOP was designed as a structured methodological guide to support more consistent reporting of US assessment procedures for limb and abdominal muscle groups by synthesizing recurring methodological issues identified in this review and organizing them into key reporting domains (Supporting Information S2: Supplementary Appendix). It encompasses key domains relevant to muscle US assessment, including physical limitations that may preclude examination, subject‐related factors such as body position and muscle condition, US probe characteristics and anatomical measurement points, image acquisition parameters, and other transparency‐relevant elements.

The SOP was externally reviewed and evaluated through pilot feasibility testing in three general intensive care units. Some of the final SOP modifications following the experts review included the introduction of an essential reporting checklist (target muscle and anatomical region, body side, body positioning, etc.) as the external experts identified that the initial version lacked a clearly defined minimum dataset required for reporting; a dedicated section describing ultrasound acquisition parameters in response to their comment that key imaging settings should be explicitly reported given the variability in ultrasound acquisition across studies; and the imaging plane, number of measurements, type of final reported value (average vs. best value), exact measurement technique (skin‐to‐bone interface), and a dedicated muscle terminology section in response to their concerns with ambiguity in measurement procedures.

The final SOP was considered feasible to implement across different ICU settings, as only minor, manageable adaptations were required, such as patient positioning and anatomical landmark identification in more complex cases. Although time constraints may represent a barrier in such situations, the involvement of two operators may improve feasibility. One operator can perform the ultrasound measurements, while the second assists with patient handling, landmark confirmation, and documentation. This approach may also enhance data quality by enabling cross‐checking of measurements. Rather than prescribing a single assessment technique, the SOP provides a structured framework for documenting methodological decisions and ultrasound acquisition parameters that may influence the interpretation, reproducibility, and comparability of muscle thickness measurements.

## 4 Discussion

The present review identified three main findings. First, although US mode, transducer type, and patient positioning were frequently reported, essential acquisition parameters such as depth, gain, and image optimization settings were inconsistently documented. Second, substantial heterogeneity was observed in anatomical landmarks, measurement criteria, and terminology, which may undermine the validity, reproducibility, and comparability of muscle thickness assessments. Third, despite their clinical relevance, upper‐limb and abdominal muscles remain underrepresented in both research and practice, limiting the comprehensive evaluation of muscle status in critically ill patients and thereby affecting decisions regarding functional recovery and rehabilitation.

Collectively, these findings highlight not only variability in assessment protocols but also important inconsistencies in methodological reporting, which may hinder interpretation of findings across studies and implementation in clinical practice. These observations provided the rationale for developing a preliminary SOP as a structured reporting framework intended to support more transparent and consistent documentation of muscle US assessment procedures across different ICU settings.

### 4.1 US Parameters and Operational Procedures

Information related to US acquisition and reporting has important implications for healthcare professionals performing muscle thickness and other ultrasound‐based measurements. Variability in ultrasound assessment procedures likely reflects differences in operator training, equipment availability, ICU logistical constraints, patient severity, and the absence of standardized acquisition and reporting frameworks across studies and institutions. Consequently, comparisons of muscle thickness trajectories between studies become challenging, particularly when acquisition parameters (including probe orientation, patient positioning, and depth/gain settings) or anatomical landmarks are incompletely described or inconsistently applied. These inconsistencies may contribute to misinterpretation of muscle atrophy and functional decline, potentially leading to inappropriate clinical decision‐making and rehabilitation planning.

For instance, US operators should consider that the flat surface of linear probes provides a higher image resolution best suited for surface structures compared to curvilinear probes used in lower‐limb muscles (Dams et al. 2024) and abdominal muscles (Gürsoy et al. 2023), which is best suited for visualizing deeper penetration (Fischetti and Scott 2007; Shriki 2014). From a practical perspective, linear transducers operating at higher frequencies may be preferable for superficial muscles that require clearer fascia delimitation and greater thickness measurement accuracy, whereas lower‐frequency or curvilinear probes may be necessary in patients with edema, obesity, or deeper abdominal muscle layers, despite lower image resolution.

Real‐time B‐mode is widely used in clinical and research settings, because of the single, perpendicular beam, whose echoes from perpendicular directions will provide reduced artifacts and less grainy images (Franchi and Narici 2023). Yet, a notable proportion of studies does not inform this basic US parameter information (Amara et al. 2022; Dams et al. 2024; Gürsoy et al. 2023; Haaksma et al. 2022; Li et al. 2020; McCaughey et al. 2019; Nguyen et al. 2023; Palakshappa et al. 2018; Parry et al. 2015; Sarwal et al. 2015; Silva‐Gutiérrez et al. 2023; Vishwas et al. 2023; Witteveen et al. 2017; Zhang et al. 2021). Failure to report basic acquisition parameters may compromise reproducibility and limit interpretation of whether observed differences in muscle thickness reflect true biological changes or methodological inconsistencies between examinations.

The US frequency and depth determine the wave penetration, thus reflecting the image resolution. Higher frequencies (e.g., 10–13 MHz) are ideal for superficial structures and greater resolution, whereas lower frequencies (e.g., 4–6 MHz) are intended for deeper muscles with lower resolution (Lawrence 2007). A broad variation of frequencies was reported regardless of the body region. Depth adjustment facilitates the US emitted to surpass physical barriers, and yet, few studies have been clear on this setting (de Moura et al. 2023; Hadda et al. 2018; Mayer et al. 2020; Sarwal et al. 2015; Silva‐Gutiérrez et al. 2023). These inconsistencies are clinically relevant because inadequate depth or frequency settings may impair visualization of muscle boundaries and contribute to measurement variability, particularly during longitudinal monitoring where small changes in muscle thickness may influence rehabilitation decisions.

Probe‐skin terminologies are often misleading by their interchangeable use in describing probe orientation or anatomical plane of acquisition. The term perpendicular should refer only to the 90‐degree angle of the US probe to the skin, while probe orientation terminology should employ transverse as reference to the short axis of the muscle being assessed, and longitudinal as reference to alignment along the long axis of the muscle, parallel orientation to the muscle fibers. Greater standardization of terminology may improve methodological transparency, facilitate replication across centers, and reduce ambiguity in interpretation of US acquisition procedures.

Studies have demonstrated that significant measurement error obscures muscle wasting trajectories in acute illness cases (Fazzini et al. 2023; Venco et al. 2024). Therefore, reliance on muscle thickness measurements alone may underestimate muscle loss, particularly when acquisition procedures are poorly standardized or when inter‐rater variability exceeds the magnitude of clinically meaningful changes in muscle size (Fivez et al. 2016; Fuest et al. 2023).

Therefore, the proposed SOP should be interpreted as a preliminary reporting framework intended to improve transparency and consistency in the documentation of US acquisition and assessment procedures rather than as a formally validated clinical standard. In practical settings, the SOP may assist physiotherapists and ICU clinicians in systematically documenting acquisition parameters, improving comparability between serial bedside assessments, and reducing reporting inconsistencies during longitudinal muscle monitoring.

### 4.2 Implications for Physiotherapy and Early ICU Rehabilitation

Abdominal, upper, and lower limb muscles play distinct roles in functional recovery and ventilatory support. Underestimation of muscle atrophy or misinterpretation of US‐derived muscle changes may lead healthcare providers of interest to misguide their decisions regarding neuromuscular impairment or functional decline (Fazzini et al. 2023). Particularly, this can implicate physiotherapists to over‐ or underly design conservative rehabilitation goals, delay mobilization, or prescribe suboptimal exercise intensity during early ICU mobilization protocols. In this context, standardized documentation of US acquisition parameters may help physiotherapists more confidently interpret serial muscle thickness changes, differentiate probable biological alterations from technical variability, and improve integration of US findings into rehabilitation planning and functional monitoring.

Abdominal muscles play essential roles in postural stabilization, airway defensive reflexes, coughing, and ventilatory compensation under increased respiratory loads (Lee et al. 2021; McCaughey et al. 2019; Shi et al. 2021). Changes in the thickness of the expiratory muscles occurred in 34% of patients and were unrelated to changes in diaphragm thickness (Shi et al. 2021); moreover, expiratory muscle atrophy has been associated with weaning failure (Schreiber et al. 2021).

Upper limb muscles, though less commonly reported (Fu et al. 2022), are essential for most activities of daily living, communication, self‐care, and mobilization in patients under mechanical ventilation (Kawahara et al. 2020; Nakanishi, Oto, et al. 2020; Nakanishi, Takashima, et al. 2020). Upper limb muscle mass loss is associated with general muscle weakness, handgrip strength, functional capacity, and specifically early decline in biceps brachii mass can be a predictor for mortality (Nakanishi, Oto, et al. 2020). Consistency for imaging procedures involving cautious probe compression on these muscle soft tissues, limb positioning for accurate muscle geometry and echogenicity evaluation, and scan modes are required to allow for early detection of wasting of these smaller muscle groups. Compared with lower‐limb assessments, upper‐limb protocols demonstrated a lower reporting frequency of reliability analyses, suggesting that these measurements in the current literature may be more vulnerable to operator‐dependent variability.

Lower limbs are easier to access, present a higher muscle loss rate, and are predominant for mobility, and load‐bearing activities compared to upper limb muscles (de Moura et al. 2023; Fu et al. 2022). Broad and nonspecific descriptions of anatomical landmarks were common in lower‐limb assessments, including terms such as “thigh muscle,” “bilateral thighs,” “femoral muscle,” or “mid‐thigh” when referring to the quadriceps muscle group (Fritsch et al. 2024; Lambell et al. 2021; Li et al. 2020). Some studies also described the quadriceps as a single muscle without clearly identifying the specific muscle examined (Hadda et al. 2018; Paris et al. 2017; Toledo et al. 2021). Nevertheless, even for the quadriceps, the most widely studied muscle group identified in this review, detailed standardization of anatomical reference points and image acquisition procedures was frequently lacking, limiting comparability across cohorts and longitudinal assessments.

Although a comprehensive ultrasound assessment of multiple muscle groups is desirable to better characterize muscle dysfunction in critically ill patients, it may not always be feasible in routine ICU practice because of time constraints, limited staffing, or patient‐related barriers. This observation was also supported during pilot feasibility testing of the proposed SOP, in which time constraints emerged as a practical challenge to standardized reporting. In such circumstances, prioritizing lower‐limb muscles, particularly the quadriceps, may represent a pragmatic approach. The rectus femoris has been widely used as a surrogate marker of whole‐body skeletal muscle mass and has been associated with ICU‐acquired weakness, functional outcomes, and longitudinal muscle loss in critically ill patients (Bald et al. 2026; Casey et al. 2022; Gu et al. 2025). The vastus intermedius may provide complementary information because of its reported associations with physical function, whereas the gastrocnemius offers a practical alternative because of its accessibility and feasibility for bedside assessment (Na et al. 2025; Parry et al. 2015; Venco et al. 2024; Zhang et al. 2021).

Nevertheless, upper‐limb and abdominal muscles should not be overlooked when rehabilitation goals involve self‐care, mobility, respiratory function, airway defense, or ventilatory support, as assessment of these muscle groups may provide clinically relevant information regarding functional impairment and recovery trajectories that would not be captured through lower‐limb assessment alone (Bald et al. 2026; Daum et al. 2026; de Carvalho et al. 2023; Fazzini et al. 2023; Formenti et al. 2019; Zhang et al. 2021). Therefore, muscle selection should be guided by the specific clinical question, patient characteristics, rehabilitation goals, and feasibility of assessment rather than by a single universal monitoring strategy.

From a practical perspective, the proposed SOP may assist physiotherapists in integrating muscle US assessment into routine ICU rehabilitation workflows by promoting systematic documentation of patient positioning, anatomical landmarks, probe characteristics, and image acquisition settings. This reporting framework is intended to standardize the documentation and communication of ultrasound‐derived muscle thickness measurements. Clinicians should apply the framework after image acquisition and measurement procedures have been completed by: (1) identifying and documenting the anatomical region and target muscle(s) evaluated using standardized muscle terminology; (2) recording the side assessed and any factors that may influence image acquisition or interpretation, such as edema, obesity, wounds, dressings, casts, or difficulties identifying anatomical landmarks; (3) reporting the anatomical landmarks and measurement sites used for image acquisition; (4) documenting muscle thickness measurements using standardized units and including reference values when available; (5) describing the imaging plane and relevant methodological details to support comparison across assessments, examiners, or clinical settings; (6) reporting any image‐quality limitations that may affect interpretation; and (7) providing a concise interpretation restricted to findings directly supported by the ultrasound assessment. This structured reporting framework may facilitate systematic communication among clinicians, support consistent monitoring of rehabilitation response over time, and improve comparability of findings across clinicians, institutions, and follow‐up assessments. In addition, pilot feasibility testing suggested that, when staffing permits, implementation of the proposed SOP may be facilitated by a two‐operator workflow, with one operator responsible for probe handling and the other for documentation and reporting.

### 4.3 Limitations and Future Directions

Some limitations acknowledged in this review include the exclusion of certain study types that may have explored the feasibility and validity of US in specific scenarios, potentially limiting a broader discussion of US applications for skeletal muscle assessment in critically ill patients. In addition, although major biomedical databases were systematically searched, databases such as Scopus and CINAHL were not included, which may have limited the retrieval of some potentially relevant studies. These restrictions were considered necessary to prioritize peer‐reviewed studies with detailed methodological reporting and feasibility within the available time constraints. Because this review aimed to characterize methodological approaches rather than synthesize clinical outcomes, no formal risk‐of‐bias assessment was conducted in accordance with PRISMA‐ScR recommendations. While this approach is appropriate for scoping reviews, it limits the ability to comment on the internal validity or comparative quality of the included studies.

Another limitation is that, as most of the analyses were qualitative in nature, some degree of interpretative bias cannot be excluded despite efforts to minimize it through pilot screening, reviewer calibration, and independent data extraction. Additionally, although the proposed SOP underwent pilot feasibility testing in ICU settings, it has not yet undergone formal evaluation of inter‐rater reliability and intra‐rater reliability or reporting consistency across different users. Therefore, the proposed SOP should not yet be interpreted as a definitive standardized protocol, but rather as a preliminary framework intended to support future methodological refinement, multicenter validation, and consensus‐building initiatives.

The strengths of this review include the comparative synthesis of US methodologies across abdominal, upper‐, and lower‐limb muscle groups in critically ill patients, with a specific focus on muscle thickness assessment. The present review comparatively examined methodological variability, reporting inconsistencies, and acquisition challenges across different muscle compartments relevant to ICU rehabilitation. Additionally, the proposed SOP was derived from recurring methodological patterns identified in the literature and externally reviewed and underwent pilot feasibility testing across different ICU environments, supporting its feasibility as a preliminary reporting framework and encouraging future multicenter validation studies. To our knowledge, this is one of the first reviews to move beyond muscle‐specific assessment protocols and propose a structured framework for documenting US procedures and acquisition settings across different muscle groups in critically ill patients.

Future investigations should evaluate the reliability, responsiveness, implementation feasibility, and clinical utility of standardized ultrasound reporting frameworks and assessment workflows across different ICU populations, professional backgrounds, and healthcare systems. Formal consensus‐building initiatives involving multidisciplinary stakeholders may also help define minimum reporting standards for muscle ultrasound studies in critical care.

## 5 Conclusions

This scoping review identified substantial variability in acquisition and reporting practices for ultrasound measurements of upper‐limb, lower‐limb, and abdominal muscle thickness in critically ill adults. Although some methodological features, such as B‐mode imaging, linear transducers, and supine positioning, were commonly reported, key methodological parameters remained inconsistently documented. Based on these findings, a preliminary SOP was developed, and subsequently refined through external expert review and pilot feasibility testing in ICU settings, as a structured reporting framework to support transparent, reproducible, and comparable reporting of muscle US assessment in critical care practice and research.

## 6 Implications of Physiotherapy Practice

The findings of this review have important implications for physiotherapists working in critical care rehabilitation. Muscle ultrasound (US) is increasingly incorporated into ICU practice to monitor muscle loss and support rehabilitation planning; however, the considerable variability identified in acquisition procedures, anatomical landmarks, measurement criteria, and reporting practices may compromise the interpretation and comparability of findings across clinicians and institutions. Greater methodological consistency in muscle US assessment is therefore essential to improve communication, longitudinal monitoring, and clinical decision‐making.

The proposed Standard Operating Procedure (SOP) provides a preliminary reporting framework that may assist physiotherapists in documenting key methodological aspects of muscle US assessment, including patient positioning, anatomical landmarks, probe characteristics, acquisition settings, and measurement procedures. Rather than prescribing a single examination protocol, the SOP is intended to improve transparency and reproducibility of US‐derived muscle thickness assessments and facilitate comparison across serial bedside evaluations and different clinical settings. Pilot feasibility testing suggested that implementation of the proposed reporting framework is feasible in ICU settings. When staffing permits, a two‐operator workflow—with one clinician responsible for probe handling and another for documentation and reporting—may facilitate integration of standardized muscle US assessment into routine physiotherapy practice.

This review also highlights that, although lower‐limb muscles remain the most frequently assessed in critically ill patients, upper‐limb and abdominal muscles should be considered whenever rehabilitation goals involve upper‐extremity function, respiratory performance, airway protection, or functional independence. Muscle selection should therefore be guided by the specific clinical question, patient characteristics, and rehabilitation objectives rather than by a single universal assessment strategy.

## Author Contributions

**Pedro Vinicius Porfirio:** conceptualization, investigation, data curation, formal analysis, writing – original draft, writing – review and editing, visualization, project administration. **Wagner Souza Leite:** conceptualization, supervision, investigation, data curation, formal analysis, validation, writing – original draft, writing – review and editing. **Layane Santana Pereira Costa:** writing – review and editing. **Pedro Henrique de Moura:** validation. **Ana Célia Oliveira dos Santos:** validation. **Emanuel Fernandes Ferreira da Silva Junior:** writing – review and editing. **Elaine Araújo de Souza:** validation. **Marismar Fernandes do Nascimento:** writing – review and editing. **Helga Cecilia Muniz de Souza:** validation. **Shirley Lima Campos:** conceptualization, project administration, data curation, supervision, writing – review and editing, funding acquisition.

## Funding

This study was supported by the Conselho Nacional de Desenvolvimento Científico e Tecnológico (CNPq) through Call No. 004/2024‐PROPESQI, Grant No. 303988/2025‐8; the Fundação de Amparo à Ciência e Tecnologia do Estado de Pernambuco (FACEPE) through Grants APQ‐1020‐4.08/25, IBPG‐0398‐2.12/23, and IBPG‐1976‐4.08/22; and the Coordenação de Aperfeiçoamento de Pessoal de Nível Superior (CAPES), Finance Code 001. Additional institutional support was provided by UFPE‐PROPG, through the PRO‐Equipamentos Program, and by PROPESQI, through the PARQUETEC Academia Program.

## Ethics Statement

The authors have nothing to report.

## Consent

The authors have nothing to report.

## Conflicts of Interest

The authors declare no conflicts of interest.

## Supporting information


Supporting Information S1

Supporting Information S2

## Data Availability

The data that support the findings of this study are available from the corresponding author upon reasonable request.
