# Supplementary material for: Methodological Approaches for Ultrasound Assessment of Peripheral and Abdominal Muscle Thickness in Intensive Care: A Scoping Review and Proposed Reporting Framework
Source: Physiother Res Int. 2026 Aug 8;31(4):e70303. doi: 10.1002/pri.70303 (PMC13452505; doi:10.1002/pri.70303)
Supplement: Supplementary file 1 [file PRI-31-e70303-s001.docx]

**Supplementary Material 1**

This material has been provided by the authors to give readers additional information about their work.

Supplement to: Methodological approaches for ultrasound assessment of peripheral and abdominal muscle thickness in intensive care: a scoping review and proposed reporting framework - Porfirio et al. (2026)

**Preferred Reporting Items for Systematic reviews and Meta-Analyses extension for Scoping Reviews (PRISMA-ScR) Checklist**

**Table S1.** PRISMA-ScR CHECKLIST ITEM

| **SECTION** | **ITEM** | **PRISMA-ScR CHECKLIST ITEM** | **REPORTED ON PAGE #** |
| --- | --- | --- | --- |
| **TITLE** | | | |
| Title | 1 | Identify the report as a scoping review. | 1 |
| **ABSTRACT** | | | |
| Structured summary | 2 | Provide a structured summary that includes (as applicable): background, objectives, eligibility criteria, sources of evidence, charting methods, results, and conclusions that relate to the review questions and objectives. | 1-2 |
| **INTRODUCTION** | | | |
| Rationale | 3 | Describe the rationale for the review in the context of what is already known. Explain why the review questions/objectives lend themselves to a scoping review approach. | 3-5 |
| Objectives | 4 | Provide an explicit statement of the questions and objectives being addressed with reference to their key elements (e.g., population or participants, concepts, and context) or other relevant key elements used to conceptualize the review questions and/or objectives. | 4-5 |
| **METHODS** | | | |
| Protocol and registration | 5 | Indicate whether a review protocol exists; state if and where it can be accessed (e.g., a Web address); and if available, provide registration information, including the registration number. | 5 |
| Eligibility criteria | 6 | Specify characteristics of the sources of evidence used as eligibility criteria (e.g., years considered, language, and publication status), and provide a rationale. | 6-7 |
| Information sources* | 7 | Describe all information sources in the search (e.g., databases with dates of coverage and contact with authors to identify additional sources), as well as the date the most recent search was executed. | 5-6 |
| Search | 8 | Present the full electronic search strategy for at least 1 database, including any limits used, such that it could be repeated. | Supplementary material |
| Selection of sources of evidence† | 9 | State the process for selecting sources of evidence (i.e., screening and eligibility) included in the scoping review. | 5-7 |
| Data charting process‡ | 10 | Describe the methods of charting data from the included sources of evidence (e.g., calibrated forms or forms that have been tested by the team before their use, and whether data charting was done independently or in duplicate) and any processes for obtaining and confirming data from investigators. | 7-9 |
| Data items | 11 | List and define all variables for which data were sought and any assumptions and simplifications made. | 7-9 |
| Critical appraisal of individual sources of evidence§ | 12 | If done, provide a rationale for conducting a critical appraisal of included sources of evidence; describe the methods used and how this information was used in any data synthesis (if appropriate). | - |
| Synthesis of results | 13 | Describe the methods of handling and summarizing the data that were charted. | 7-9 |
| **RESULTS** | | | |
| Selection of sources of evidence | 14 | Give numbers of sources of evidence screened, assessed for eligibility, and included in the review, with reasons for exclusions at each stage, ideally using a flow diagram. | 9 |
| Characteristics of sources of evidence | 15 | For each source of evidence, present characteristics for which data were charted and provide the citations. | 9 and supplementary Material |
| Critical appraisal within sources of evidence | 16 | If done, present data on critical appraisal of included sources of evidence (see item 12). | - |
| Results of individual sources of evidence | 17 | For each included source of evidence, present the relevant data that were charted that relate to the review questions and objectives. | 9-14 and supplementary Material |
| Synthesis of results | 18 | Summarize and/or present the charting results as they relate to the review questions and objectives. | 9-14 and supplementary Material |
| **DISCUSSION** | | | |
| Summary of evidence | 19 | Summarize the main results (including an overview of concepts, themes, and types of evidence available), link to the review questions and objectives, and consider the relevance to key groups. | 15-16 |
| Limitations | 20 | Discuss the limitations of the scoping review process. | 22 |
| Conclusions | 21 | Provide a general interpretation of the results with respect to the review questions and objectives, as well as potential implications and/or next steps. | 20-22; and 24 |
| **FUNDING** | | | |
| Funding | 22 | Describe sources of funding for the included sources of evidence, as well as sources of funding for the scoping review. Describe the role of the funders of the scoping review. | 25 |

JBI = Joanna Briggs Institute; PRISMA-ScR = Preferred Reporting Items for Systematic reviews and Meta-Analyses extension for Scoping Reviews.* Where *sources of evidence* (see second footnote) are compiled from, such as bibliographic databases, social media platforms, and Web sites.

† A more inclusive/heterogeneous term used to account for the different types of evidence or data sources (e.g., quantitative and/or qualitative research, expert opinion, and policy documents) that may be eligible in a scoping review as opposed to only studies. This is not to be confused with *information sources* (see first footnote). ‡ The frameworks by Arksey and O’Malley (6) and Levac and colleagues (7) and the JBI guidance (4, 5) refer to the process of data extraction in a scoping review as data charting*.* § The process of systematically examining research evidence to assess its validity, results, and relevance before using it to inform a decision. This term is used for items 12 and 19 instead of "risk of bias" (which is more applicable to systematic reviews of interventions) to include and acknowledge the various sources of evidence that may be used in a scoping review (e.g., quantitative and/or qualitative research, expert opinion, and policy document).

*From:* Tricco AC, Lillie E, Zarin W, O'Brien KK, Colquhoun H, Levac D, et al. PRISMA Extension for Scoping Reviews (PRISMAScR): Checklist and Explanation. Ann Intern Med. 2018;169:467–473. [doi: 10.7326/M18-0850](http://annals.org/aim/fullarticle/2700389/prisma-extension-scoping-reviews-prisma-scr-checklist-explanation).

**Database Search Strategy**

### Embase (Elsevier, Embase.com)

I

('ultrasound'/exp OR ultrasound OR 'ultrasonography'/exp OR ultrasonography) AND ('skeletal muscle'/exp OR 'peripheral muscle' OR 'quadriceps muscle layer thickness' OR 'lower limb muscle layer thickness' OR 'abdominal muscle layer thickness' OR 'rectus femoris muscle thickness' OR 'biceps brachii muscle thickness' OR 'abdominal muscle'/exp OR 'abdominal muscle') AND ('intensive care unit'/exp OR 'intensive care unit')

1.744 results

### PubMed (National Library of Medicine, NCBI)

#1

(("Ultrasonography"[Mesh] OR ultrasound OR ultrasonography) AND ("Muscle, Skeletal"[Mesh] OR "peripheral muscle" OR "quadriceps muscle layer thickness" OR "lower limb muscle layer thickness" OR "abdominal muscle layer thickness" OR "rectus femoris muscle thickness" OR "biceps brachii muscle thickness" OR "abdominal muscle")) AND("Intensive Care Units"[Mesh] OR "intensive care unit"))

506 results

### BIREME (Organização Pan-Americana da Saúde / Organização Mundial da Saúde, OPAS/OMS)

#1

("ultrasound" OR "ultrasonography" OR "thickness") AND ("peripheral muscle" OR "quadriceps muscle layer thickness" OR "lower limb muscle layer thickness" OR "abdominal muscle layer thickness" OR "rectus femoris muscle thickness" OR "biceps brachii muscle thickness" OR "abdominal muscle") AND ("intensive care unit")

43 results

**Description of ultrasonography evaluation.**

**Table S2.** Description of ultrasonography evaluation of upper limb muscles.

| Author (year), country | Description of Ultrasonography-Assessed Muscle Thickness of upper limb muscles - body and probe position, ultrasound settings informations and thickness criteria for measurement |
| --- | --- |
| de Arruda et al. (2025), Brazil | **Body position:** Supine, with patient’s head of bed placed at 30-degree angle.  **General US operational info:** High-frequency linear-array transducer.  **Probe positioning landmarks**: Biceps brachii: transducer was positioned at two-thirds of the craniocaudal distance between the acromion and the cubital fossa.  **Thickness measurement**: Not reported. |
| Silva Barbosa et al. (2023), Brazil | **Body position:** Supine, in a semi-recumbent position with patient’s head of bed placed at 30-degree angle, with arms and legs extended and muscles in a relaxed state.  **General US operational info:** B-mode, with a multifrequency linear array transducer, frequency of 8.5 MHz, depth of 7.4 cm and gain of 64 db (constant image acquisition settings)  **Probe positioning landmarks**: Biceps brachii: transducer perpendicularly along the axis of the arm on its anterior surface at the point located between the lower third and the upper two-thirds of the distance between the acromion and the cubital fossa. Forearm flexor group: transducer positioned between the upper two-fifths and the lower three-fifths of the distance from the antecubital crease to the distal end of the radius.  **Thickness measurement**: Biceps brachii: between the upper part of the humerus and the superficial fascia of the biceps (including the brachialis muscle). Forearm flexor group: between the interosseous membrane located close to the radius and the superficial fascia of the flexors. |
| Moura et al. (2023), Brazil | **Body position:** Supine, with bed’s heat at 30 degrees.  **General US operational info:** B-mode, with a linear array transducer, frequency of 8.0 MHz, depth of 3.5cm. Time gain and compensation were set to the neutral position. The focus number and area were maximally increased to remain consistent across all participants and to adjust for differences in muscle size between them.  **Probe positioning landmarks**: Deltoid: 1/2 between the acromion and deltoid tuberosity with the upper limb in anatomical position and supported on the stretcher. The author also assessed the biceps brachii using the protocol by Witteveen et al. (2017), included in this review.  **Thickness measurement**: The selected region was set to include as much muscle as possible, excluding surrounding bone or fascia. |
| Chapple et al. (2022), Australia | **Body position:** Supine, with legs relaxed lying flat in extension with toes facing the roof.  **General US operational info:** B-mode, frequency of 5–13 MHz. All settings such as gain, focus and contrast were kept constant. Depth was set to 6 cm for quadriceps and mid-arm measures and 4.2 cm for forearm measures. The transducer was held perpendicular to the skin surface to capture a transverse (cross-sectional) image.  **Probe positioning landmarks**: Biceps brachii: humerus lateral epicondyle and superior lateral acromion border and at the anterior forearm at the mid-point between the tip of the olecranon and ulna styloid process  **Thickness measurement:** Not reported. |
| Lambel et al. (2021), Australia | **Body position:** Supine, with bed’s heat at approximately 30 degrees.  **General US operational info:** B-mode, with a multifrequency linear array transducer and frequency of 4–12 MHz. The probe was held perpendicular to the skin, and depth was adjusted to visualize the relevant bone.  **Probe positioning landmarks**: Mid-upper arm: A mark was made on the skin at the midway point between the tip of the acromion and the olecranon process. The thickness of the bicep flexor compartment was imaged with the elbow extended and the forearm supinated and resting on the bed. Forearm: A point was marked at 30% proximal between the ulnar styloid process and the head of the radius. With the hand supinated and the forearm relaxed on the bed, the image was taken.  **Thickness measurement**: Mid-upper arm: from the subcutaneous adipose tissue–muscle interface to the muscle-bone interface of the humerus. Forearm: the distance between the subcutaneous adipose tissue–muscle interface and muscle-bone interface of the ulna. |
| Mayer et al. (2020), USA | **Body position:** Not reported; arms are supported in passive extension and neutral rotation.  **General US operational info**: Linear array transducer and frequency of 7.5-MHz. Constant depth of acquisition set at 4.9 cm.  **Probe positioning landmarks**: Biceps brachii: transducer was placed perpendicular to the long axis of the arm on its anterior surface two-thirds of the distance from the acromion process to the antecubital crease.  **Thickness measurement**: Not reported. |
| Hadda et al. (2018), India | **Body position:** Supine.  **General US operational info:** B-mode, with a linear array probe, frequency of 5.0–13.0 MHz and depth set at 4.9 cm.  **Probe positioning landmarks:** A circumferential mark was applied midway between the greater tuberosity and the tip of the olecranon process of the humerus. Linear array USG probe was placed on the anterior aspect of this circumferential line, perpendicular to the skin, and the probe was moved along the line drawn till a suitable image was obtained. A point corresponding to the center of the probe was marked with a vertical line, and this point was taken as the reference point for all the subsequent measurements. Arm muscle thickness was measured while the upper limb relaxed, lying parallel to the body, the elbow extended and the palm facing the ceiling.  **Thickness measurement:** Between superficial fat-muscle interface and periosteum. |
| Witteveen et al. (2017), The Netherlands | **Body position:** Not reported.  **General US operational info**: Linear array transducer, frequency of 4–13 MHz and constant image acquisition settings. Depth settings were adapted, if needed.  **Probe positioning landmarks**: Biceps brachii: 2/3 distance from acromion to antecubital crease, transducer positioned on humerus located in the middle and neurovascular bundle on the edge of the picture. Flexor carpi radialis: 1/3 distance from antecubital fold to distal radius (muscle has triangular shape), transducer positioned horizontally and flat position of radius and ulna. 15 ̊ angle between radius and ulna.  **Thickness measurement**: Biceps brachii: from humerus to upper border of BB (including brachialis muscle). Flexor carpi radialis (FCR): From deepest point of FCR to upper border of FCR. |
|  | |

**Table S3.** Description of ultrasonography evaluation of lower limb muscles.

| Author (year), country | Description of Ultrasonography-Assessed Muscle Thickness of lower limb muscles - body and probe position, ultrasound settings informations and thickness criteria for measurement |
| --- | --- |
| de Arruda et al. (2025), Brazil | **Body position:** Supine, with patient’s head of bed placed at 30-degree angle.  **General US operational info:** High-frequency linear-array transducer.  **Probe positioning landmarks**: Rectus femoris: lower middle third of the distance between the anterosuperior iliac spine and upper edge of the patella.  **Thickness measurement**: Not reported. |
| Dams et al. (2024), Belgium | **Body position:** Supine, in full extension and relaxed position of the right leg (dominant/non-dominant side mentioned).  **General US operational info:** Curvilinear array probe with a frequency range of 5-2MHz was used. Depth was adjusted to view both muscle layers and femur.  **Probe positioning landmarks**: Quadriceps: transducer positioned at the middle point between the greater trochanter and the proximal border of the patella.  **Thickness measurement**: Not reported. |
| Fritsch (2024), Germany | **Body position:** Not reported.  **General US operational info:** B-mode, with a convex transducer and frequency of 5.0–13.0 MHz.  **Probe positioning landmarks**: On the top of the femur perpendicular to the femur, creating a transverse section through the femur; on the half as well as between the lower and the two upper thirds of a line between the upper edge of the patella and the spina iliaca anterior superior. Thigh muscle thickness was measured while the leg resting straight, the knee extended and the great toe facing the ceiling  **Thickness measurement**: The distance between the upper edge of the femur and the uppermost muscle fascia. |
| Gürsoy et al. (2023), Turkey | **Body position:** Supine.  **General US operational info:** Linear probe (US operational info not reported).  **Probe positioning landmarks**: Rectus femoris muscle: probe was placed at a right angle to the dorsal thigh by determining the midpoint between the thoracantary major and the patella proximal border.  **Thickness measurement**: Rectus femoris muscle: In the axial ultrasound image, the muscle was visualized under the subcutaneous adipose tissue, and its thickness was measured. |
| Silva Barbosa et al. (2023), Brazil | **Body position:** Supine, in a semi-recumbent position with patient’s head of bed placed at 30-degree angle, with arms and legs extended and muscles in a relaxed state  **General US operational info:** B-mode, with a multifrequency linear array transducer, frequency of 8.5 MHz, depth of 7.4 cm and gain of 64 db (constant image acquisition settings)  **Probe positioning landmarks**: Quadriceps femoris: transducer positioned perpendicularly to the muscle at the point located two-thirds of the distance between the anterior superior iliac spine and the superior surface of the patella on the anterior thigh. Tibialis anterior muscle: transducer positioned perpendicularly to the long axis of the muscle at the point between the upper quarter and lower three-quarters of the distance from the lower border of the patella to the tip of the lateral malleolus.  **Thickness measurement**: Quadriceps femoris: between the upper part of the femur and the superficial fascia of the rectus femoris (includes the rectus femoris and vastus intermedius); Tibialis anterior: between the interosseous membrane located close to the tibia and the superficial fascia of the tibialis anterior. |
| Moura et al. (2023), Brazil | **Body position:** Supine, with bed’s heat at 30 degrees.  **General US operational info:** B-mode, with a linear array transducer, frequency of 8.0 MHz, depth of 3.5cm. Time gain and compensation were  set to the neutral position. The focus number and area were maximally increased to remain consistent across all participants and to adjust for differences in muscle size between them.  **Probe positioning landmarks**: Tibialis anterior: 1/3 between the edge of the patella and lateral malleolus with the lower limb in knee extension (neutral position) and supported on the stretcher. Gastrocnemius (medial and lateral): 1/3 between the popliteal surface and insertion of the Achilles tendon with the lower limb in triple flexion and foot resting on the bed. The author also assessed the quadriceps using the protocol by Witteveen et al. (2017), included in this review.  **Thickness measurement**: The selected region was set to include as much muscle as possible, excluding surrounding bone or fascia. |
| Silva-Gutiérrez et al. (2023), Chile | **Body position:** Supine, with the head at 30°, ankles in a neutral position and upper and lower limbs extended.  **General US operational info:** Linear transducer with frequency of 6-13 MHz.  **Probe positioning landmarks**: Anterosuperior iliac spine and the superior border of the patella delimited the midpoint between this distance.  **Thickness measurement**: Vastus intermedius: between the cortical bone and the fascia that separates the rectus femoris from the vastus intermedius. Rectus femoris: from the fascia that separates the rectus femoris from the vastus intermedius to the beginning of the superior fascia of the rectus femoris; Quadriceps: through the internal border of the epimysium from the cortical bone to the fascia top of the rectus femoris. Measurements were recorded in cm. |
| Rajagopal et al. (2023), India | **Body position:** Supine.  **General US operational info:** B-mode, with a linear transducer with frequency of 5–10 MHz.  **Probe positioning landmarks**: Rectus femoris muscle: straight line was drawn from the anterior inferior iliac spine to the patella on the right leg and the 2/3rd point was measured using a caliper and marked with a permanent marker. A transducer was placed perpendicular to the long axis of the muscle (i.e., perpendicular to the major axis of the limb) and the transverse view of the anterior thigh.  **Thickness measurement**: From the internal borders of the rectus femoris muscle to the external border of the same (in cm). |
| Nguyen et al. (2023), France | **Body position:** Supine.  **General US operational info:** Linear array transducer (US operational info not reported).  **Probe positioning landmarks:** Rectus femoris: approximately three-fifths of the distance from the anterior superior iliac spine to the superior patellar border.  **Thickness measurement**: Not reported. |
| Chapple et al. (2022), Australia | **Body position:** Supine, with legs relaxed lying flat in extension with toes facing the roof.  **General US operational info:** B-mode, frequency of 5–13 MHz. All settings such as gain, focus and contrast were kept constant. Depth was set to 6 cm for quadriceps and mid-arm measures and 4.2 cm for forearm measures. The transducer was held perpendicular to the skin surface to capture a transverse (cross-sectional) image.  **Probe positioning landmarks**: Vastus intermedius and rectus femoris: i) at the border between the lower third and upper two-thirds between the Anterior Superior Iliac Spine and the upper pole of the patella, and ii) at the midpoint between the Anterior Superior Iliac Spine and the upper pole of the patella.  **Thickness measurement:** Not reported. |
| Andrade-Junior et al. (2021), Brazil | **Body position:** Not reported.  **General US operational info:** B-mode.  **Probe positioning landmarks**: Referenced to Seymour et al. (2009).  **Thickness measurement**: Thickness of the anterior compartment of the quadriceps muscle (rectus femoris and vastus intermedius, in cm) |
| Lambell (2021), Australia | **Body position:** Supine, with bed’s heat at approximately 30 degrees.  **General US operational info:** B-mode, with a multifrequency linear array transducer and frequency of 4–12 MHz. The probe was held perpendicular to the skin, and depth was adjusted to visualize the relevant bone.  **Probe positioning landmarks**: Bilateral thighs: With knees extended and relaxed, a point was marked at the anterior superior iliac spine and the upper pole of the patella. A point was then marked at the midpoint and two-thirds point between these landmarks.  **Thickness measurement**: From the subcutaneous adipose tissue–muscle interface to the muscle-bone interface of the femur at both points on both thighs. |
| Zhang et al. (2021), China | **Body position:** Supine position with extended elbows, wrists, knees and relaxed muscles, meanwhile the palms and toes of patients were facing or pointing to the ceiling.  **General US operational info:** Linear array transducer, with frequency of 10–13 MHz.  **Probe positioning landmarks**: Vastus intermedius and rectus femoris: the second third of the distance between the anterior inferior iliac spine, and the midpoint of the proximal border of the patella for rectus femoris, and the midline of the same distance as rectus for vastus intermedius.  **Thickness measurement**: Not reported. |
| Toledo et al. (2021), Brazil | **Body position:** Supine, in semi-recumbent position (30-45°) and knees extended.  **General US operational info:** B-mode, with linear array probe and a multifrequency of 5.0-13.0 MHz. Depth was adjusted to obtain the image of the femur.  **Probe positioning landmarks**: Quadriceps: border of the lower third and upper two-thirds between the anterior superior iliac spine and the upper part of the patella.  **Thickness measurement**: Distance from the upper margin of the femoral bone and the lower border of the superficial fascia of the rectus femoris muscle, including both the rectus femoris and vastus intermediaries muscles. |
| Li et al. (2020), China | **Body position:** Supine, with bed’s heat at approximately 30 degrees and the lower limbs were naturally relaxed and straightened.  **General US operational info:** Linear probe with a frequency of 6-13MHz.  **Probe positioning landmarks**: Femoral muscle: The probe coated with enough coupling is placed vertically at 1/3 of the upper and lower edges of the iliac upper and patella of the patient's right lower limb, and the probe position is adjusted to allow the image to display both the straight femur and the intermediate femur.  **Thickness measurement**: The thickness of the muscle is the inner vertical distance between the echoes. It is the vertical distance between the middle muscle membranes above the femur. |
| Mayer et al. (2020), USA | **Body position:** Supine, with legs supported in passive extension and neutral rotation.  **General US operational info:** B-mode, with linear array transducer, frequency of 7.5-MHz and constant depth of acquisition set at 4.9 cm.  **Probe positioning landmarks**: Quadriceps femoris: transducer was placed perpendicular to the muscle at two-thirds the distance from the anterior-superior iliac spine to the superior aspect of the patella on the anterior thigh. Tibialis anterior: transducer placed perpendicular to the long axis of the muscle at one-fifth the distance from the head of the fibula to the tip of the lateral malleolus.  **Thickness measurement**: Not reported. |
| Palakshappa et al. (2018), USA | **Body position:** Not reported.  **General US operational info:** Linear transducer with 5–10 MHz.  **Probe positioning landmarks**: Transducer placed perpendicular to the long axis of the thigh; at two-thirds of the distance between the anterior superior iliac spine and the superior patellar border was marked with a skin marker.  **Thickness measurement**: Quadriceps (rectus femoris-vastus intermedius complex): the linear distance from below the subcutaneous tissue down to the level of the femur. |
| Hadda et al. (2018), India | **Body position:** Supine.  **General US operational info:** B-mode, with a linear array probe, frequency of 5.0–13.0 MHz and depth set at 4.9 cm.  **Probe positioning landmarks**: A circumferential mark was applied at the midway between the tip of the greater trochanter and the lateral joint line of the knee. Linear array USG probe was placed on the anterior aspect of this circumferential line, perpendicular to the skin, and the probe was moved along the line drawn till a suitable image was obtained. a point corresponding to the center of the probe was marked with a vertical line and this point was taken as a reference point for all the subsequent measurements.  **Thickness measurement**: Between superficial fat-muscle interface and periosteum. |
| Witteveen et al. (2017), Netherlands | **Body position:** Not reported  **General US operational info**: Linear array transducer, frequency of 4–13 MHz and constant image acquisition settings. Depth settings were adapted, if needed.  **Probe positioning landmarks**: Rectus femoris: halfway from anterior superior iliac spine and superior border of patella. Transducer positioned on the femur located in the middle with vastus intermedius and rectus femoris visible above the femur. Tibialis anterior: 1/3 distance from inferior border of patella to the lateral malleolus; transducer positioned on tibial cortex at about 45 ̊ angle. Central fascia is evident.  **Thickness measurement**: Rectus femoris: from femoral bone to upper border of rectus femoris (including vastus intermedius muscle). Tibialis anterior: from lower border of tibialis anterior to upper border |
| Paris et al. (2017), Canada | **Body position:** Supine, knees extended and relaxed.  **General US operational info:** B-mode, with a multifrequency linear array transducer**.**  **Probe position on landmarks:** Quadriceps: anterior surface of the quadriceps from the midpoint between the anterior superior iliac spine and the upper pole of the patella and the border of the lower third and upper two-thirds between the anterior superior iliac spine and the upper pole of the patella.  **Thickness measurement:** The distance between the upper margin of the femoral bone and the lower boundary of the deep fascia of the rectus femoris, incorporating both the rectus femoris and the vastus intermedialis. |
| Parry et al. (2015), Australia | **Body position:** Supine, with their knee in passive extension and neutral rotation;  **General US operational info:** Linear transducer, with frequency of 8.5-MHz.  **Probe positioning landmarks**: Anterior imaging for rectus femoris and vastus intermedius: transducer placed perpendicular to the long axis of the anterior thigh, two-thirds distance from the anterior superior iliac spine to the superior patella border; Lateral imaging for vastus lateralis: 5 cm laterally from the first imaging point. The lateral image was obtained in an extended field of view mode over a distance of 10 cm running in a caudal-cephalad direction.  **Thickness measurement**: Not reported. |
| Sarwal et al. (2015), USA | **Body position:** Supine, with the leg supported in passive extension and neutral rotation.  **General US operational info:** Linear array transducer with frequency of 8.5-MHz and 5-cm depth on the right lower limb.  **Probe positioning landmarks:** Rectus femoris muscle**:** placed perpendicular to the long axis of the thigh on its anterior surface, two-thirds of the distance from the anterior superior iliac spine to the superior patellar border.  **Thickness measurement:** The maximum thickness of the rectus femoris–vastus intermedius complex from the femur to subcutaneous tissue**.** |

**Table S4.** Description of ultrasonography evaluation of abdominal muscles.

| Author (year), country | Description of Ultrasonography-Assessed Muscle Thickness of abdominal muscles - body and probe position, ultrasound settings informations and thickness criteria for measurement |
| --- | --- |
| Gürsoy et al. (2023), Turkey | **Body position:** Supine.  **General US operational info:** Curvilinear probe (US operational info not provided).  **Probe positioning landmarks**: Rectus abdominis: probe was placed in the transverse plane just lateral to the umbilicus.  **Thickness measurement**: Between the layers of the anterior abdominal wall and measured bilaterally. |
| Moura et al. (2023), Brazil | **Body position:** Supine, with bed’s heat at 30 degrees.  **General US operational info:** B-mode, with a linear array transducer, frequency of 8.0 MHz, depth of 3.5cm. Time gain and compensation were  set to the neutral position. The focus number and area were maximally increased to remain consistent across all participants and to adjust for differences in muscle size between them.  **Probe positioning landmarks**: Rectus abdominis: 2-3 centimeters above the umbilicus. Oblique (external and internal) and transverse abdominis: Perpendicular line between the anterior superior iliac spine and umbilical line.  **Thickness measurement**: The selected region was set to include as much muscle as possible, excluding surrounding bone or fascia. |
| Vishwas et al. (2023), India | **Body position:** Not reported.  **General US operational info:** Linear probe (US operational info not reported).  **Probe positioning landmarks**: Rectus abdominis: place the linear ultrasound probe in a horizontal fashion about 3 cm above the umbilicus and 3 cm away from the midline; Internal oblique, external oblique, and transversus abdominis: place the linear ultrasound probe in a horizontal fashion in the anterior axillary line, at the midpoint between the costal margin and the iliac crest.  **Thickness measurement**: Not reported. |
| Nguyen et al. (2023), France | **Body position:** Half-seated.  **General US operational info:** Linear array transducer (US operational info not reported).  **Probe positioning landmarks:** Expiratory muscle: approximately two-thirds of the distance between the umbilic and the anterosuperior iliac spine at the end of inspiration  **Thickness measurement**: Not reported. |
| Amara et al. (2022), India | **Body position:** Not reported.  **General US operational info:** Linear probe (US operational info not reported).  **Probe positioning landmarks:** Rectus abdominis**:** probe was placed horizontally about 2–3 cm above the umbilicus and 2–3 cm away from the midline. Internal oblique, external oblique, and transversus abdominis: probe was placed horizontally at the anterior axillary line, approximately at the mid-point of the right costal margin and the iliac crest.  **Thickness measurement**: Not reported. |
| Haaksma et al. (2022), Netherlands | **Body position:** Supine.  **General US operational info:** Linear transducer, with frequency of 4–12 MHz. Gain and depth settings were freely adjustable by the operator.  **Probe positioning landmarks**: Rectus abdominis: examined on the right side, perpendicular to the abdominal wall at its thickest portion on the right side of the patient, slightly supraumbilical. Lateral abdominal muscles: examined on the right side, perpendicular to the abdominal wall approximately between at the midpoint of arcus costalis and ilium.  **Thickness measurement:** Rectus abdominis: Calipers were placed between the fascia surrounding the muscle. Lateral abdominal muscles: the most ventral and dorsal fascia in addition to measurement of individual muscle thickness. Muscle thickness at end inspiration (for rectus abdominis and lateral wall muscles). |
| Lambell et al. (2021), Australia | **Body position:** Supine, with bed’s heat at approximately 30 degrees.  **General US operational info:** B-mode, with a multifrequency linear array transducer and frequency of 4–12 MHz. The probe was held perpendicular to the skin, and depth was adjusted to visualize the relevant bone.  **Probe positioning landmarks**: Abdominals: A mark was made 3 cm to the right of the umbilicus. The probe was rotated, and the image was saved as the rectus abdominis muscle was positioned horizontally on the screen.  **Thickness measurement**: From the distance between the upper and lower inner muscle fascia layers (in the center of the image). |
| Yang et al. (2021), China | **Body position:** Supine.  **General US operational info:** B mode, linear transducer with frequency of 5.0-12.0 MHz.  **Probe positioning landmarks**: Rectus abdominis: the probe was placed perpendicularly on the upper abdominal wall, the upper abdomen was scanned from the level of the umbilicus to 5 cm cephalad, the probe was tilted until both sheaths of the muscle were seen clearly on the monitor. When both sheaths of the muscle were clearly seen, the image was frozen for thickness measurement.  **Thickness measurement**: The distance between sheaths of rectus abdominis (in cm). |
| McCaughey (2019), Australia | **Body position:** Not reported.  **General US operational info:** Not reported.  **Probe positioning landmarks:** Expiratory muscle: approximately two-thirds of the distance between the umbilic and the anterosuperior iliac spine at the end of inspiration.  **Thickness measurement**: Not reported. |
| Dall’ Acqua et al. (2017), Brazil | **Body position:** Supine, with bed’s heat at approximately 30 degrees.  **General US operational info:** B-mode, linear-array transducer, with 3.5-mm and frequency of 7.5 MHz.  **Probe positioning landmarks**: Rectus abdominis: at a lateral distance of 2 cm from the umbilicus.  **Thickness measurement**: Between the inner edge of the upper and lower aponeuroses of the rectus abdominis. |

**Summary of studies included in the scoping review on ultrasonographic assessment of muscle thickness in critically ill adults.**

**Table S5.** Description, reliability data, and study findings on Ultrasonography-Assessed Muscle Thickness in Adult Critically Ill Patients.

| **Author (year), country** | **Sample characteristics** | **Muscles or areas of the body** | **Main outcomes** | **Training** | **Reliability data** |
| --- | --- | --- | --- | --- | --- |
| de Arruda et al. (2025), Brazil | N. of participants: 43  Age: 45  Sex: NR  Inclusion criteria: Patients aged ≥18 years, with acute brain injuries within 48 h of hospitalization and at least five days of ICU stay | Biceps brachii and the rectus femoris | Neurocritical patients experience significant muscle deterioration during hospitalization. | Researcher experienced in peripheral muscle ultrasound and trained in image acquisition | NR |
| Sundarsingh et al. (2025), India | N. of participants: 120  Age: 75  Sex: M - 62.5%  Inclusion criteria: Patients aged ≥65 years admitted to the ICU and expected to stay for more than 24 h | Rectus Femoris and Vastus Intermedius | There is an independent association between Quadriceps Muscle Layer Thickness and frailty. | Trained examiner | NR |
| Dams et al. (2024), Belgium | N. of participants: 29  Age: 60.9  Sex: M - 66%  Inclusion criteria: Patients under MV with confirmed COVID-19 disease and anticipated to be intubated >72 h | Quadriceps and diaphragm | Muscle atrophy and decline in muscle strength appear in the earliest stages after admission to the ICU and are related to functional outcome. | NR | The authors reported that they conducted intra- and inter-operator reliability tests, but they did not present the statistical results |
| Fritsch et al (2024), Germany | N. of participants: 50  Age: 64.5  Sex: M - 64%  Inclusion criteria: Patients under MV and with an expected treatment period of at least 5 days in the weaning unit. | Quadriceps femoris and diaphragm | Diaphragmatic MT increased over the course of prolonged weaning, while the diameter of a representative peripheral muscle decreased. | Trained examiner under supervision of a physician with several years of experience | NR |
| Gürsoy et al. (2023), Turkey | N. of participants: 43  Age: 67.51  Sex: M - 72.1%  Inclusion criteria: Patients who were hospitalized in Muğla Training and Research Hospital Anesthesia ICU. | Rectus abdominis and rectus femoris | Rectus abdominis and rectus femoris MT measured by US can be a reliable and easily applicable quantitative method that can be used to determine nutritional risk in the ICU. | One ICU specialist | NR |
| Nguyen et al. (2023), France | N. of participants: 263 / 47  Age: 66 / 74  Sex: M - 64% / 70%  Inclusion criteria: Patients admitted on surgical ICU. | External and internal oblique, transversus abdominis, quadriceps, rectus femoris and vastus intermediate | US expiratory abdominal MT was associated with 28-day mortality, supporting its use in predicting ICU patient outcome. | All US measurements were performed by trained physicians who practice point-of-care US on a daily basis. | NR |
| Rajagopal et al. (2023), India | N. of participants: 50  Age: 56.4  Sex: M - 66%  Inclusion criteria: Patients admitted to the ICU, with an expected length of stay of at least 7 days and willing to consent were enrolled in the study. | Rectus femoris | The study demonstrates that there is significant muscle wasting in critically ill patients and this positively correlates with the severity of illness. | NR | NR |
| Silva Barbosa et al. (2023), Brazil | N. of participants: 10  Age: 62  Sex: M - 60%  Inclusion criteria: ≥ 18 years old and who were admitted to the ICU. | Biceps brachii, forearm flexor group, quadriceps femoris, tibialis anterior and diaphragm | Excellent intra-examiner reliability for echogenicity and inter-examiner reliability for thickness were found in all muscle groups. For MT intra-examiner reliability, excellent results were found with a “good” correlation in one diaphragm assessment. Excellent inter- and intra-examiner reliability of the thickness assessment and intra-examiner echogenicity of all muscles analyzed were found. | Four health professionals trained by an expert Ph.D Physiotherapist. Three of them were well-trained and experienced, with more than 5 years of experience working in ICU, and the fourth was ending her two-year residency program. | Excellent inter-examiner (ICC: 0.778–0.942) and intra-examiner (ICC: 0.798–0.988) |
| de Moura et al. (2023), Brazil | N. of participants: 30  Age: 59.8  Sex: M - 70%  Inclusion criteria: Patients under MV for more than 48 h and had a confirmed diagnosis of COVID-19 by RT‒PCR. | Quadriceps, rectus femoris, vastus intermedius, tibialis anterior, medial and lateral gastrocnemius, deltoid, biceps brachii, rectus abdominis, internal and external oblique, and transversus abdominis | Peripheral and abdominal muscle mass loss is progressive over the ICU time of mechanically ventilated patients with COVID-19. The lower limbs, left quadriceps and right rectus femoris muscles showed greater muscle wasting during the first week. | A single assessor with a master’s degree in physical therapy and years of experience in ultrasound assessment in critically ill patients. His training consisted of technical lectures and individual training sessions from a formal ultrasound physiotherapist/researcher specialized in clinical musculoskeletal ultrasound. | The authors reported that they performed a pilot study with intra-assessor ICC and limits of agreements strong and reliable, but did not present the statistical results. |
| Silva-Gutiérrez et al. (2023), Chile | N. of participants: 132  Age: 59.5  Sex: M - 85%  Inclusion criteria: Patients under MV for more than 48 h and had a confirmed diagnosis of SARS-CoV-2. | Quadriceps (rectus femoris and vastus intermedius) | Critically ill patients infected with SARS-CoV-2 and requiring MV presented decreased muscle mass, strength, and mobility during their ICU stay. | Evaluator with more than 3 years of experience in measuring MT using clinical US | NR |
| Vishwas et al. (2023), India | N. of participants: 81  Age: 55.70  Sex: M - 64.2%  Inclusion criteria: Patients under MV in ICU who met the criteria for spontaneous breathing trial for the first time for weaning ventilation. | Rectus abdominis, internal oblique, external oblique, and transversus abdominis | The mNUTRIC score is an independent predictor of thinner abdominal expiratory muscles in mechanically ventilated critically ill patients. | NR | NR |
| Amara et al. (2022), India | N. of participants: 81  Age: 55.7  Sex: NR  Inclusion criteria: Patients under MV and were put on spontaneous breathing trial for the weaning process | Rectus abdominis, internal oblique, external oblique, and transversus abdominis | Abdominal expiratory muscles were thicker in patients with simple weaning compared to those with difficult or prolonged weaning. The pattern of expiratory MT followed rectus abdominis > internal oblique > external oblique > transversus abdominis in both simple weaning and difficult or prolonged weaning groups. | NR | NR |
| Haaksma et al. (2022), Netherlands | N. of participants: 30  Age: 69  Sex: M - 19%  Inclusion criteria: Patients admitted to the ICU who tested positive for SARS-CoV-2 and were ventilated for < 24 h. | External and internal oblique, transversus abdominis, rectus abdominis and diaphragm | In ventilated subjects with COVID-19, subjects with decreased or unchanged thickness had a longer ventilation time than those with increased thickness. Neuromuscular-blocking agent use was associated with decreased thickness. Rectus MT did not change over time, whereas lateral abdominal MT decreased but this change was not statistically significant. | 5 well-trained researchers with extensive ultrasound experience (> 2 y of almost daily ultrasound use). | The authors reported that they did not perform reproducibility measurements as these had already been conducted in previous studies |
| Chapple et al. (2022), Australia | N. of participants: 42 / 38  Age: 58.2 / 53.5  Sex: 60% / 66%  Inclusion criteria: Pattients ≥ 18 years old and receiving invasive MV | Quadriceps (vastus intermedius and rectus femoris) and bicep  brachii | Augmented calorie delivery may not attenuate muscle loss or recovery of strength or function 6  months post-ICU. | Two trained operators. | NR |
| Andrade-Junior et al. (2021), Brazil | N. of participants: 32  Age: 64.1  Sex: M - 93.8%  Inclusion criteria: ≥ 18 years old SARS-CoV-2 infection confirmed by RT-PCR, ICU stay and the absence of lower limb amputation. | Anterior compartment of the quadriceps muscle (rectus femoris and vastus intermedius). | In ICU patients with severe COVID-19, muscle wasting and decreased muscle strength occurred early and rapidly during 10 days of ICU stay with improved mobility and respiratory functions, although they remained below normal levels. | NR | NR |
| Lambell et al. (2021), Australia | N. of participants: 50  Age: 52  Sex: M - 38%  Inclusion criteria:Patients with a CT scan including the L3 area ≤24 hours before or ≤72 hours after ICU admission. | Mid-upper arm (bicep flexor compartment), forearm (ulna MT), abdominal (rectus abdominis) and thighs. | Ultrasound shows potential for assessing muscularity at ICU admission. | Two trained investigators | Intrarater CV = 2.2% and ICC = 0.998, and interrater CV = 3.6% and ICC = 0.992 |
| Toledo et al. (2021), Brazil | N. of participants: 74  Age: 62.3  Sex: M - 54.1%  Inclusion criteria: Patients admitted to the ICU and expecting to need MV for at least 48h. | Quadriceps | The measurement of quadriceps MT using ultrasound showed that critically ill patients on mechanical ventilation presented with muscle wasting and greater loss of MT was associated with worse outcomes. | Four operators who were trained in using bedside US in the ICU | NR |
| Yang et al. (2021), Taiwan | N. of participants: 27  Age: 67.9  Sex: M - 59%  Inclusion criteria: Patients transferred to ICU after abdominal procedures. | Rectus abdominis | The body mass index-rectus abdominis thickness ratio is related to the outcomes of patients transferred to ICU after major abdominal surgery. | NR | NR |
| Zhang et al. (2021), China | N. of participants: 13 / 24  Age: 48.92 / 68.38  Sex: M - 69.2% / 54.2%  Inclusion criteria: Patients with an anticipated ICU stay of at least 2 days. | Biceps brachii, rectus femoris and vastus intermedius | US changes in muscles presented good diagnostic accuracy. | Two researchers trained and qualified | The correlation coefficients of measurement accuracy of the two researchers were 0.88, 0.90, and 0.91 for biceps brachii, rectus femoris, and vastus intermedius, respectively. |
| Li et al. (2020), China | N. of participants: 45  Age: 58  Sex: M - 55%  Inclusion criteria: Patients with estimated duration of MV ≥ 24 h and no organic lesions of the brain. | Quadriceps femoris (rectus femoris and vastus intermedius). | Bedside ultrasound measures the thickness of quadriceps muscle can identify ICU-AW early. | Two healthcare professionals. | NR |
| Mayer et al. (2020), USA | N. of participants: 6  Age: 55,67  Sex: F - 66,7%  Inclusion criteria:Patients admitted to the medical ICU OR patients with a long-term stay quantified as >21 days following an acute critical illness (recovery) OR healthy individuals in an outpatient rehabilitation gym that were not being treated for active musculoskeletal conditions. | Biceps brachii, quadriceps femoris, and tibialis anterior muscles | Excellent interrater reliability was observed regardless of the level of experience, severity of patient illness, or patient setting. These findings indicate that the use of muscle US by physical therapists can accurately capture reliable images in patients with a range of illness severity and different clinical practice settings across the continuum of care. | Five sonographers were included in this study: 4 inexperienced “novice” sonographers and 1 experienced “expert” sonographer (pulmonary and critical care physician, MD., with >10 years of experience in muscle US acquisition and analysis | Intraclass correlation coefficient values ranged from 0.76 to 0.97 with an average for all raters and all muscles of 0.903, indicating excellent reliability of image acquisition. |
| McCaughey et al. (2019), Australia | N. of participants: 10 / (CG) 10  Age: 56.5 / 61  Sex: M - 70% / 50%.  Inclusion criteria: Patients dependent on MV due to critical illness. | Rectus abdominis, internal and external oblique and transversus abdominis | The study demonstrates the feasibility of using abdominal FES with critically ill mechanically ventilated patients. | NR | NR |
| Hadda et al. (2018), India | N. of participants: 70  Age: 55.91  Sex: M - 64.3%  Inclusion criteria: Patients admitted under Pulmonary Medicine services with a diagnosis of sepsis with medical illness. | Flexor compartment (biceps brachii and coracobrachialis) and the extensor compartment (quadriceps muscles) | Critically ill patients with sepsis exhibit a gradual decline in MT of both the arm and thigh. Decline in MT was associated with in-hospital mortality. US has a potential to identify patients at risk of worse in-hospital and post-discharge outcomes. | A single operator who was trained in using bedside US in ICU. | NR |
| Palakshappa et al. (2018), USA | N. of participants: 19  Age: 66  Sex: M - 63%  Inclusion criteria: Patients admitted to the medical ICU at the Hospital of the University of Pennsylvania between June and December 2015 with a diagnosis of sepsis. | Quadriceps | Muscle atrophy as detected by the rate of change in rectus femoris cross-sectional area moderately correlated with strength one week after sepsis admission. | One experienced critical care physician (trained by a fellowship-trained musculoskeletal radiologist) obtained all of the images. A subset of the images was reviewed with the radiologist to ensure accurate image capture. | NR |
| Dall' Acqua et al. (2017), Brazil | N. of participants: 11 / 14.  Age: 56 / 61.  Sex: M - 63% / 64%.  Inclusion criteria: Patients who had been hospitalized for no longer than 15 days and had received at least 24 h of invasive MV. | Rectus abdominis, pectoralis major and diaphragm. | Thickness of rectus abdominis and pectoralis muscles were preserved in the intervention group (p < 0.001), whereas a significant reduction of thickness in the control group was observed (p > 0.001). No difference in diaphragm thickness was observed. | One highly experienced professional (4 years) | ICC for the test-retest muscle was r = 0.996 for the chest muscle, r = 0.998 for the rectus abdominis, and r = 0.972 for the diaphragmatic MT. |
| Witteveen et al. (2017), The Netherlands | N. of participants: 30 / 41  Age: 62 / 60  Sex: M - 73% / 61%  Inclusion criteria: Newly admitted ICU patients, who were under MV for ≥ 48 h. | Biceps brachii, flexor carpi radialis, rectus femoris and tibialis anterior | Neuromuscular ultrasound does not discriminate between patients with and without ICU-AW at the time the patient awakens and is therefore not able to reliably diagnose ICU-AW in ICU patients relatively early in the disease course | Trained assessors | NR to MT (only echo intensity) |
| Paris et al. (2017), Canada | N. of participants: 149  Age: 59  Sex: M - 57.7%  Inclusion criteria: Patients with a cross-sectional abdominal CT scan of at least the L3 vertebra performed for clinical reasons <24 hours before or <72 hours after ICU admission. | Quadriceps | The results suggest that quadriceps MT alone with the protocol of the study may not accurately identify patients with low muscle mass | NR | Intrarater CV of 7% and ICC ranged from 0.93 to 1.00, with an average ICC of 0.98 and CV of 7%; and interrater CV = 13% and ICC = 0.94 |
| Parry et al. (2015), Australia | N. of participants: 22  Age: 56  Sex: M - 59%  Inclusion criteria: Patients expected to under MV for > 48 h and remain in the ICU for at least 4 days. | Quadriceps | Muscle wasting occurs rapidly in the ICU setting. US is a useful surrogate measure for identifying future impairment. Vastus intermedius may be an important muscle to monitor in the future because it demonstrated the greatest change in muscle quality and had the strongest relationship to volitional measures. | One experienced assessor | NR |
| Sarwal et al. (2015), Australia | N. of participants: 40  Age: NR  Sex: NR  Inclusion criteria: Patients admitted to the neurocritical care unit for diaphragm images and to a mixed surgical-medical ICU for quadriceps. | Diaphragm and quadriceps | Excellent interobserver reliability exists regardless of expertise level for quantitative analysis of muscle parameters on sonography in the critically ill population. | Two assessors were experienced sonographers (neurocritical care physician and senior ICU physiotherapist) and two were novice users (exercise physiology students) | Very good interobserver reliability between all assessors for measurement of MT regardless of technique for both diaphragm and quadriceps muscles, with ICC values ranging from 0.84 to 0.99. |
| M: male; F: female; MT: muscle thickness; MV: mechanical ventilation; ICU: intensive care units; US: ultrasonography; NR: not reported; CG: control group; ICC: intraclass correlation coefficient; CV: coefficient of variation; CT: computed tomography; mNUTRIC: Modified Nutrition Risk in Critically ill; NRS-2002: Nutritional Risk Screening 2002; SARS-CoV-2: severe acute respiratory syndrome coronavirus 2. | | | | | |
