# Supplementary material for: Methodological Approaches for Ultrasound Assessment of Peripheral and Abdominal Muscle Thickness in Intensive Care: A Scoping Review and Proposed Reporting Framework
Source: Physiother Res Int. 2026 Aug 8;31(4):e70303. doi: 10.1002/pri.70303 (PMC13452505; doi:10.1002/pri.70303)
Supplement: Supplementary file 2 [file PRI-31-e70303-s002.docx]

**Standard Operating Procedure for reporting ultrasound acquisition and assessment procedures for peripheral and abdominal muscle thickness in critically ill patients.**

Porfirio et al. (2026), Physiotherapy Research International

| **MUSCLES TERMINOLOGY** | |
| --- | --- |
|  Upper arm | Deltoid, biceps brachii and/or brachialis |
|  Forearm | Brachioradialis, flexor carpi radialis, flexor carpi ulnaris, and/or flexor digitorum superficialis/ profundus |
|  Trunk | Pectoralis major, external oblique, internal oblique, rectus abdominis and/or transversus abdominis |
|  Thigh | Rectus femoris, vastus intermedius, vastus lateralis, and/or vastus medialis |
|  Lower leg | Tibialis anterior and/or gastrocnemius (medial and lateral heads) |
| **REPORTING ITEMS** | |
| The US report should contain sufficient methodological information to allow interpretation, reproducibility, and comparison across serial assessments, examiners, institutions, and research studies. The following items should be documented: | - Target muscle(s) and anatomical region assessed; - Body side evaluated; - Patient positioning; - Anatomical landmarks and measurement site(s); - Probe characteristics, including transducer type and frequency range; - Ultrasound acquisition settings, including imaging mode and any relevant parameters (e.g., depth and gain); - Measurement procedure, including imaging plane (transverse or longitudinal), number of measurements obtained, and method used to derive the final value (e.g., single measurement or average of repeated measurements); - Muscle thickness values, reported in standardized units (mm or cm); - Factors potentially affecting image acquisition or interpretation; - Examiner identification and level of ultrasound training or expertise. |
| **ADDITIONAL INFORMATION** | |
| **Potential exam limitations** | - Excessive adiposity tissue, severe edema, casts, dressings, open wounds/ulcers, inability to visualize internal intra-articular, bone, or fascia landmarks. |
| **Subject or patients factors** |  |
| Position of subject | - Supine, lying with head of the bed elevated to 30º; |
| Use of supports | - Resting on the stretcher for more stable positioning; |
| Rotation of limbs | - Forearms supinated with hand palms upwards and/or legs relaxed and therefore slightly rotated outwards; |
| State of muscle | - Relaxed; |
| Muscle name | - Use the terminology in reference to reporting your target muscles for US measurements. |
| **US probe factor** | - B-mode; - Linear probe with high frequency (6-12 MHz) for superficial muscles; - Linear probe with low frequency (2-5 MHz) for deep muscles. |
| **Measuring points** |  |
| Finding the measuring points | - Palpation of precise anatomical landmarks; |
| Measuring points | - Reference to Supplementary Material to description for the muscle of interest; |
| Labelling | - Removable paint marker pen; |
| Side | - Dominant; |
| Additional information | - Avoid skin regions with wound or edematous; obese patients may need more support or vary body positions. |
| **Evaluation of US scan** |  |
| Probe position | - Long axis or short axis, and perpendicular (probe angle to the skin); |
| Compression | - Minimal compression without losing gel/skin contact; |
| Plane | - Verification of convex shape of skin and blurred borders in US scans; |
|  | - At each point, 1 scan in the short-axis plane and 1 scan in the long-axis plane; |
| Repetition | - Unlimited (except for reliability assessment); |
| What is measured? | - Muscle thickness of target muscle; |
| How is it measured? | - Muscle thickness measured at the exact center of the scan drawing the shortest possible line from skin to bone surface. |
| Additional information | - In case of difficult visualization for edema: |
|  | a) start to spot muscle tissue from the bone surface and see how far muscle goes upwards; b) compare short-axis and long axis plane to better identify muscle fascia; and c) compare both right and left body sides - one side may be prone to less edema, which can facilitate fascia identification. |

Illustration adapted from smart.servier.com.
